# An Interdisciplinary Flipped Classroom Module on Postpartum Depression Using Telemedicine and Online Teaching

**DOI:** 10.1007/s40596-021-01475-2

**Published:** 2021-05-10

**Authors:** Erika M. Monasch, Paula M. Wadell, Sara Baumann, Melissa Hopkins, Melody Y. Hou

**Affiliations:** University of California, Davis School of Medicine, Sacramento, CA USA

Postpartum depression, a subtopic of Behavioral Health: Abnormal Processes: Mood Disorders in the integrated United States Medical Licensing Examination (USMLE) Content Outline [1], presents a unique collaborative teaching opportunity between psychiatrists and obstetrician-gynecologists (OBGYN). A pre-clerkship educational session that efficiently illustrates clinical relevance of basic science coursework could prepare students to see patients with postpartum depression in both their psychiatry and OBGYN clerkships. In a traditional flipped learning format, students prepare individually and asynchronously prior to class with content provided by their instructors. Students then come together in-person for a synchronous learning session during which students apply the concepts they studied and teach and learn from their peers [2]. However, the coronavirus disease of 2019 (COVID-19) pandemic [3] required many medical schools to move to virtual pre-clerkship curricula [4], and adapt various methods such as videoconferencing, online standardized patient encounters, and digital flipped classroom sessions [5–7]. We present here a postpartum depression educational module that adapts traditional flipped learning [2, 8] to a videoconferencing platform and introduces pre-clerkship students to the use of telemedicine for postpartum depression.

## Approach

We (3 psychiatrists, 1 OBGYN, and 1 medical student) created a 110-min flipped classroom module on postpartum depression for the pre-clerkship psychiatry course in the 2nd-year medical student curriculum at our institution for the 2020–2021 academic year. Although originally planned as an in-person session, our school switched to online remote instruction due to the COVID-19 pandemic 2 months prior to its scheduled date. Due to this transition, we chose to make this session non-mandatory.

We adapted our pre-clerkship postpartum depression flipped classroom case from a peer-reviewed OBGYN clerkship postpartum depression team-based learning session published by Freerksen et al. in 2015 [9]. Our students cover endocrinology and reproduction, including labor and lactation but not postpartum depression, during their first year. Pre-class preparation included an Association of Professors in Gynecology and Obstetrics (APGO) Basic Sciences video on postpartum depression [10] reviewing the neurobiological mechanisms underlying postpartum depression, changes in steroid hormones in the postpartum period, and the pharmacology of selective serotonin reuptake inhibitors (SSRIs), as well as a psychiatry review article by Stewart and Vigod [11] which discusses pathophysiology, treatment, and therapeutics for postpartum depression. We adapted the published individual readiness assessment test from Freerksen et al. [9] into a 9-item readiness self-assessment quiz to review these materials (quiz available upon request). Students could retake the quiz as many times as necessary to achieve the 80% passing score to participate in the flipped classroom module. We required all students to take this quiz to receive credit, although attendance in the group session was not required. We recorded simulated 2- and 4-week postpartum video visits through the Zoom videoconferencing platform between a standardized patient and a psychiatry instructor to be played during the session. We conducted the flipped classroom module as a synchronous online session on Zoom. Students could download the session’s case presentation before the session to take notes. The main psychiatry instructor led the discussion while the other facilitators from both psychiatry and OBGYN contributed their expertise in the discussion and monitored the classroom chat window. After reviewing the self-assessment quiz answers, we began the case presentation. After showing the 2-week postpartum visit video, we divided students into breakout rooms of eight students to work on pre-determined open-ended questions regarding differential diagnosis and management of postpartum depression, based on Freerksen et al.’s work (questions available upon request) [9]. The course coordinator rotated the facilitators through the breakout rooms to answer questions. After 15 min of work, students returned to the larger discussion room to discuss answers and advance the case. We did the same following the 4-week postpartum visit video before ending the session.

We assessed students’ perceptions after the flipped classroom module via a voluntary, anonymous online survey, which was exempted by our Institutional Review Board. Completion of the survey implied consent to study participation. We sent an invitation to all students enrolled in the course to participate in the survey with reminders until 2 weeks following the session.

## Outcomes

Of the 123 second-year medical students at the University of California, Davis School of Medicine, 116 (94.3%) attended this non-mandatory flipped classroom module. We recorded student attendance over the course of the session using the Zoom webinar attendee report: 116 students participated in the readiness self-assessment quiz review, of which 97 participated in the first breakout session, 84 in the following large group discussion, 68 in the next breakout session, and 67 in the final large group discussion. At the beginning of the session, some students expressed confusion about whether the session was mandatory, which we clarified was not. Of the students who attended the session, 78% (91/116) responded to our survey. Most students felt that the preparation materials were helpful (75/91, 82%), that the pre-class quiz helped them assess their preparation for the session (80/91, 88%), and that the session met its learning objectives (83/89, 93%). Eighty-nine percent of students (80/90) agreed or strongly agreed that having instructors from both OBGYN and psychiatry in the session improved their understanding of postpartum depression, and most students found the peer discussion in the breakout rooms helpful for their learning (68/89, 76%). Students agreed or strongly agreed that the video visits helped them understand how to interview patients with postpartum depression (71/89, 80%) and how clinicians use telemedicine in patient care (71/90, 79%). Students largely felt that this session should be included in next year’s course (81/90, 90%).

Our survey included two open-ended questions asking students what they found most useful about the flipped classroom module and how the session could be improved for future years. Over half of the respondents cited the video visits as most useful (29/50, 58%), followed by having instructors from both OBGYN and psychiatry present (11/50, 22%). Students also appreciated learning about postpartum depression (7/50, 14%), working in breakout groups (5/50, 10%), and preparing for the session via the review article (3/50, 6%) and the readiness self-assessment quiz (2/50, 4%). Several students (11/32, 34%) felt the breakout rooms could be improved, with most of these students suggesting that a facilitator be assigned to each breakout room. Some students also recommended changes to the self-assessment quiz (5/32, 16%) or cited technical difficulties (3/32, 9%). Three students requested more clarity about the optional nature of the session (9%), and three students wished that the psychiatry course’s lecture on depression had been available to view beforehand (9%). Finally, students advocated for using more gender-inclusive language (2/32, 6%) and for more case examples, real patient cases, or live patients (3/32, 9%).

## Interpretation

The COVID-19 pandemic has significantly impacted medical school curricula, requiring educators to deliver pre-clerkship coursework virtually through various methods [4–6]. We used Zoom videoconferencing to deliver a multi-disciplinary, pre-clerkship flipped classroom module that taught second-year medical students about postpartum depression and exposed them to telemedicine. Overall, students found this session helpful in learning about postpartum depression. Study limitations include student attrition over the duration of the module to 57.8% at the end of the session, and the low response rate (50 students) to the open-ended feedback questions.

Efforts to teach about postpartum depression have focused on training medical students during their clinical clerkships in psychiatry [12] or OBGYN [9] using standardized patient cases or in-person flipped classroom sessions. Another module introduced postpartum mood disorders to pre-clerkship medical students via a discussion of a short story on postpartum psychosis [13]. We feel that our flipped classroom session for pre-clerkship students is an excellent teaching module on postpartum depression that is adapted to a socially distanced learning environment. Additionally, our module was developed in collaboration between psychiatrists and OBGYNs, who have different clinical perspectives on the same disorder. We had multiple meetings over several months to map out each specialty’s resources to form a cohesive plan, and students appreciated the interdisciplinary nature of our approach. Students found the telemedicine encounter with a psychiatry instructor and a standardized patient particularly helpful in improving their understanding of how a psychiatrist and patient interact through clinical telemedicine. With this session, our students may feel better prepared to recognize and care for patients with postpartum depression during their clerkships.

The use of Zoom highlighted the differences between remote breakout rooms and traditional in-person flipped classroom sessions, the latter requiring only a few facilitators who can roam between student groups as needed. In the Zoom format, students desired more facilitator time in the breakout rooms to increase student engagement, which requires more facilitators. Although Zoom attendance showed attrition over the course of the session, we do not feel that attendance should be required during a pandemic. Non-mandatory, Zoom-based coursework can be more engaging than in-person coursework and may promote student comfort and autonomy during this stressful time [14]. Other improvements to the session would include positioning this session to follow the students’ lecture on depression [15], writing the readiness self-assessment quiz items in the National Board of Medical Examiners (NBME) format [16], and adding answer explanations to the quiz platform to preserve time during the active session for clinical application rather than quiz review.

Dramatic changes in healthcare delivery have resulted from the COVID-19 pandemic. Specifically, physicians’ use of telemedicine and team-based care is greatly expanding, and medical students need to be prepared earlier to develop the skills necessary to deliver high-quality collaborative care online [17]. Our flipped classroom module represents an engaging, multi-disciplinary, online educational experience to teach pre-clerkship students about postpartum depression and telemedicine.
